# Fabrication and Characterization of Non-Equilibrium Plasma-Treated PVDF Nanofiber Membrane-Based Sensors

**DOI:** 10.3390/s21124179

**Published:** 2021-06-18

**Authors:** Quazi Nahida Sultana, Mujibur Khan, Rajib Mahamud, Mohammadsadegh Saadatzi, Papia Sultana, Tanvir Farouk, Rafael Quirino, Sourav Banerjee

**Affiliations:** 1 Department of Mechanical Engineering, Georgia Southern University, Statesboro, GA 30460, USA; qn00123@georgiasouthern.edu (Q.N.S.); ps02154@georgiasouthern.edu (P.S.); 2 Department of Mechanical Engineering, University of South Carolina, Columbia, SC 29208, USA; rajib.mahamud@gmail.com (R.M.); saadatzi@email.sc.edu (M.S.); TFAROUK@sc.edu (T.F.); banerjes@cec.sc.edu (S.B.); 3 Department of Chemistry, Georgia Southern University, Statesboro, GA 30460, USA; rquirino@georgiasouthern.edu

**Keywords:** PVDF nanofiber, electrospinning, non-thermal, plasma, sensors, piezoelectricity

## Abstract

The effect of a self-pulsing non-equilibrium plasma discharge on piezoelectric PVDF nanofiber membrane was investigated. The plasma discharge was generated in air with a DC power source, with a discharge current of 0.012 mA, a nominal interelectrode separation of 1 mm, and discharge voltage of ~970 V. In a continuous fabrication process, the electrospinning method was used to generate thin nanofiber membrane with a flow rate of 0.7–1 mL h^−1^ and 25–27 kV voltage to obtain the nanofiber with high sensitivity and a higher degree of alignment and uniformity over a larger area. Plasma treatment was applied on both single layer and multi-layer (three layers) nanomembranes. In addition, simultaneously, the nanofiber membranes were heat-treated at a glass transition temperature (80–120 °C) and then underwent plasma treatment. Fourier-transform infrared (FTIR) spectroscopy showed that the area under the curve at 840 and 1272 cm^−1^ (β phase) increased due to the application of plasma and differential scanning calorimeter (DSC) indicated an increase in the degree of crystallinity. Finally, PVDF sensors were fabricated from the nanofibers and their piezoelectric properties were characterized. The results suggested that compared to the pristine samples the piezoelectric properties in the plasma and plasma-heat-treated sensors were enhanced by 70% and 85% respectively.

## 1. Introduction

Polyvinylidene fluoride (PVDF) is a widely studied polymer that exists in non-linear, piezo-, and pyroelectric forms with potential applications in a variety of sensors and actuators. Due to its low cost, resistance to chemicals, oxidation, and UV radiation, as well as its favorable mechanical properties, PVDF is used in pressure sensors, bimorph actuators, microphones, gas flow, and humidity sensors, as well as electro-mechanical and ultrasonic sensors. It is also successfully used as a substrate for sensing flexural waves in plates [1,2,3,4,5,6]. PVDF exists in a combination of four crystalline phases, α, β, γ, and δ. Due to the highest remnant polarization and specific chain conformation, the β phase in PVDFs has attracted more attention than other pyro and piezoelectric materials [7]. 

The relative quantity of each phase is dependent on the thermal, mechanical, and electrical processing conditions used to produce the PVDF films. PVDF is composed of repeated units of fluorinated hydrocarbon connected linearly; α-phase is the most common naturally available phase. PVDF is composed of polymer chains that occur in non-polar trans-gauche-trans-gauche (TGTG) conformation. While α-phase PVDF is non-polar and non-piezoelectric, β-phase PVDF is highly oriented with polymer chains in all-trans zigzag conformation. Hence, β-phase is responsible for the piezo- and pyroelectric properties of the polymer [7]. Applying mechanical deformation by stretching an α-PVDF converts it to a β-PVDF. Uni-axial or bi-axial stretching provides the alignment of the molecular chain, and the application of a strong electric field at an elevated temperature allows the dipole alignment along the direction of the applied electric field [8,9,10]. It was found that, besides mechanical stretching, there are various other methods that could enhance the piezoelectricity of the PVDF membranes. In this work, the possibility of an atmospheric pressure non-equilibrium thermal plasma was explored.

Non-equilibrium, atmospheric pressure plasmas have gained significant interest for the activation and functionalization of a wide range of materials [11,12,13,14,15,16]. Plasma-treated polymers in air or oxygen media exhibits higher surface energy. Low pressure or vacuum plasmas were applied in treating polymer surfaces [17,18], and results reported show an increase in surface energy, adhesive bond strength, and wettability [19]. It is reported that the activation of the polyethylene terephthalate (PET) and polyethylene naphthalate (PEN) surfaces with atmospheric pressure helium–oxygen plasma, was due to the formation of C−O and C=O bonds. The application of low-power (~120 W) atmospheric plasmas on PVDF electrospun nanofiber breaks the C-H bonds and forms C=O, C−O, and hydrophilic groups, and retains the C−F bonds [20]. The plasma interaction can also lead to better chain orientation at the surface layer and increase crystallinity related to the electroactive β phase. On the other hand, the treatment of PVDF fiber using high-power plasma (240–500 W) that typically has higher temperature can result in C−F bond scission that leads to a decrease in the β phase [21]. It should be noted that the traditional plasma treatment methods of polymers utilize glow discharges that have low discharge current, high electric field, and are highly non-equilibrium. In this study, a self-pulsing “sub-normal” glow discharge was investigated which has transient variations in discharge current and voltage/electric field. As the crystallization process is strongly driven by the electric field, the “sub-normal” plasma is expected to increase the ordering of the β phase. The “sub-normal” plasma discharge belongs to the nonequilibrium plasma class that was characterized by extremely low gas temperature (~350–400 K) and a high electron temperature (~4 eV; 1 eV = 11,604 K). While the discharge is governed by electric field and radical deposition, the effect of gas heating is minimum. To determine the effect of heat, the PVDF nanofiber samples were simultaneously plasma- and heat-treated. The β-phase content in plasma-treated and plasma-heat-treated samples were further evaluated using Fourier-transform infrared (FTIR) spectroscopy and differential scanning calorimeter (DSC).

PVDF has a wide range of applications such as different types of sensors in medical diagnostics, wearable systems, structural health monitoring, electromechanical equipment, etc. [22,23,24,25]. Some of the major applications of PVDF technologies include PVDF-LiPF_6_ as a piezo-electrolyte for harvesting/storing the vibration mechanical energy of human motion for wearable electronics [26], CuO/PVDF nanoarray piezo-anode for realizing an integrated self-charging power cell (SCPC) [27], and the piezoelectrically-driven electrochemical process involved in SCPC [28]. However, the principal disadvantage of PVDF is its piezoelectric coefficients. They are significantly lower than most common piezoelectric materials (e.g., piezoelectric ceramics). Therefore, the effect of β-phase content on the magnitude of the output signals and on the overall piezoelectric electro-mechanical coupling requires further investigation. It has been established that the piezoelectric response improves as the β-phase content in the specimen increases. Therefore, to evaluate the piezoelectric responses from the proposed plasma-treated PVDF nanofibers, a unique characterization setup was developed. In the characterization process, first the plasma-treated fibers were used to fabricate sensors embedding the fibers between two copper electrode thin films, and then the piezoelectric coefficients of the fabricated sensors were measured and determined using the newly developed impact test setup.

## 2. Experimental Details

Polyvinylidene fluoride (PVDF) powder (molecular weight 534,000 g/mol), and N,N-dimethyl-formamide (DMF) were procured from Sigma–Aldrich (Sigma-Aldrich, St. Louis, MO, USA) where DMF was used as a solvent. For Pure PVDF fiber, PVDF was dissolved in a DMF solvent by an overhead stirrer. PVDF was added by 15 wt% for electrospun nanofiber membrane. To get a homogenous mixture, an overhead stirrer was used for 3–4 h at high speed and the PVDF was added progressively to dissolve in the DMF solvent completely. Pure PVDF nanofiber membranes were manufactured from the prepared solution using an electrospinning process.

### 2.1. Electrospinning Setup

**Figure 1:**
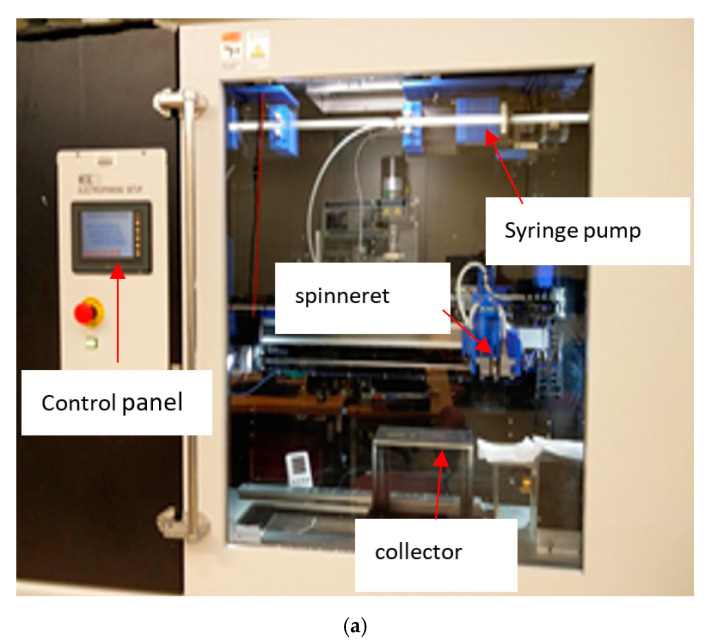

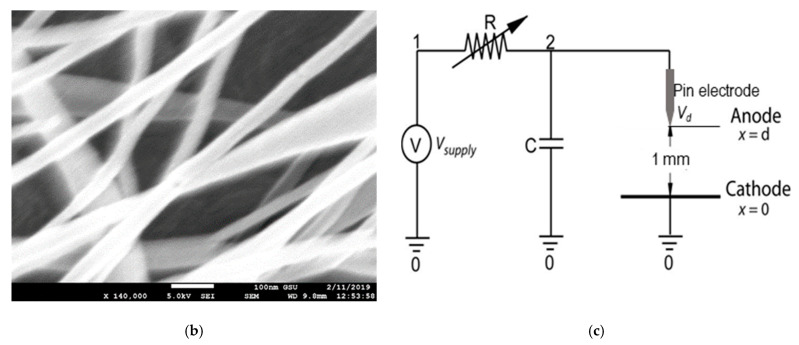
(**a**) Picture of the electrospinning unit (**b**) SEM image of PVDF nanofiber (**c**) schematic of the pin to plate plasma discharge driving circuit, with numbers representing the different circuit the nodes. V_s_: Power supply, connecting node 1–0. R: Resistor, connecting node 1–2 (3 MΩ). C_p_: Parasitic capacitance, present intrinsically in the system due to external cables and connections. V_d_: Plasma discharge, connecting node 2–0.

The fabrication was performed using a MECC NF-500 electrospinning system (Figure 1a). Two feed pumps in the system were added for a controlled feeding rate (0.2–10.0 mL h^−1^) of the polymer solutions at the spinneret nozzle. The spinneret assembly could be modified to include either a single-nozzle or coaxial nozzle setup. The metallic body of the spinneret was connected to the positive terminal of a high voltage direct current (DC) power supply with a maximum voltage rating of 60 kV. The ground terminal of the power supply was connected to the collector plate placed at a distance below the spinneret. The distance between the spinneret nozzle tip and the collector plate can be adjusted to modify the electric field strength between the terminals. An aluminum foil was used to cover the grounded plate collector, which functioned as a conductive substrate for deposition of the nanofibers during electrospinning. The process parameters—voltage, spinneret tip-to-collector distance (TCD)—and feed rates were adjusted by using the control unit of the NF-500 system. The inner chamber of the NF-500 unit was connected to a dehumidifier unit which maintains constant humidity level during the electrospinning process.

### 2.2. Characterization

The fabricated fibers were characterized by DSC and FTRI spectroscopy. DSC tests were performed with a Mettler Toledo TA Q100 (Mettler-Toledo, Columbus, OH, USA) DSC under a nitrogen atmosphere. The temperature range was from 0 to 350 °C with a heating rate of 3 °C min^−1^. The real-time heating curves were generated using the Thermal Advantage Universal Analysis2000 V4.5A (TA Instruments, New Castle, DE, USA) software package. The morphology of the fibers was studied using Fourier transform infrared spectroscopy (FTIR). The FTIR spectra was obtained in the range of 600–4000 cm^−1^ using a Thermo Nicolet Avatar 370 Fourier-transformer (FTIR) instrument (LabX, Midland, ON, Canada). Fiber filaments were placed across the diamond crystal using a high-pressure clamp, and the fiber was pressed to the crystal surface, and then a background spectrum was collected. The background spectrum contained information about the molecules of gases and solvents. To get the information for the sample, the background spectrum was subtracted from the sample spectrum. All spectra were collected at 4 cm^−1^ spectral resolution and 100 scans were added. The KnowItAll TM software was used to analyze all spectra.

### 2.3. Electrospinning Parameters

The single nozzle spinneret assembly housed a 4-mL syringe loaded with the polymer solution. Figure 1a shows the electrospinning system with voltage control, a syringe pump, a spinneret, and the collector for the electrospinning process, whereas the SEM image, shown in Figure 1b, represents the PVDF nanofiber samples with diameters between 50 and 120 nm. A 16-gauge needle was attached to the tip of the syringe through a metallic luer lock connector. The plunger of the spinneret was traversed at the selected feed rate by the feed pump drive through a hydraulic piston mechanism. In the experiment, the flow rate and the voltage were adjusted to obtain the optimum operating condition of electrospinning process. The applied flow rate was 0.9 mL h^−1^ and the voltage was set at 25 kV. The spinneret traversed at a 15-cm stroke length, and the distance between the syringe needle and the collector was kept at 160 mm. The electrospinning was conducted at room temperature, with a relative humidity of ~40%, and was carried out for 3 h to produce the PVDF nanofiber membrane. 

### 2.4. Self Pulsing “Sub-Normal” Glow Discharge and Application on PVDF Film

A dc-driven glow discharge plasma operating at atmospheric pressure air was used to treat the PVDF samples. However, unlike the traditional stable glow discharge, this specific discharge operated in the “sub-normal” discharge. A “sub-normal” glow discharge temporally fluctuated between a corona and normal glow continuously [15,29] and possessed the characteristics of both a corona and normal glow discharge. It had a very low current and therefore minimized the thermal effect but at the same time has higher electric field due to the sharp temporal and spatial variations. For treating the PVDF films, this “sub-normal” glow discharge was generated between a powered pin-type electrode (i.e., anode) and a grounded plate (i.e., cathode) where the sample to be treated was placed. An inter-electrode separation distance of ~1 mm was maintained between the two electrodes. A schematic of the driving circuit is presented in Figure 1c. The powered pin electrode was connected to the Spellman DC power supply (Model: SL20P2000). The self-pulsing “sub-normal” discharge had a pulsing frequency of 20 Hz, discharge voltage of V_rms_ = 960 V, and discharge current of I_rms_ = 0.012 mA. When the plasma discharge was formed, the ionized heavy particles were accelerated towards the grounded plane. The low conductivity of the PVDF sample allowed a surface charge build-up on the sample surface. The discharge also had a temporally varying strong electric field. Both the surface charge and the strong external electric field contributed to the poling of the PVDF sample.

For PVDF electrospun nanofiber membrane and PVDF film, samples were heated at 100 °C, which is above the glass transition temperature of PVDF. Afterwards, the samples were plasma-treated for an hour.

### 2.5. Sensor Fabrication

**Figure 2:**
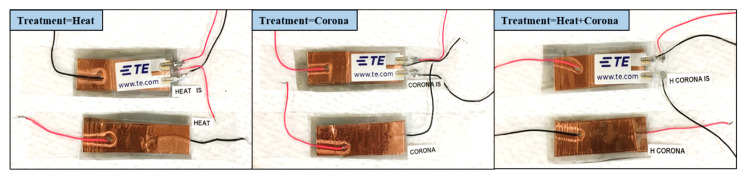
Images of the different fabricated sensors using the fabricated, plasma, and plasma-heat treated samples.

Bare PVDF nanofiber membranes, plasma, and plasma-heat-treated PVDF membranes were further used to fabricate application driven sensors. Sensors were fabricated to sense direct impact and responses were recorded. The first the PVDF nanofiber membranes were cut up to desired shapes and sizes. The membranes were then laminated, and sandwiched between 30-µm thick silicone layer at 65 °C. Then thin layers of adhesive copper tape were used to create reliable electrical connections to provide potential and ground terminals. Two sensors from each type of PVDF that received different treatments were fabricated. Figure 2 shows images of the fabricated sensors. The first category of sensors utilized off-the-shelf pressure sensors sandwiched on the top surface of each sensor to measure the exact applied force on the fabricated samples. In the second category, sensors were made with required copper electrodes and wirings. The proposed sensors were designed to sense pressure and force from an impact event.

### 2.6. Impact Test Setup

After fabrication of different sensors with and without pressure sensors, the PVDF sensors were subjected to standardized impact tests to measure the piezoelectric effect. After an impact on the sensors, the sensor bent downwards, causing tension at the bottom surface and compression on the top surface. However, this situation was not static but dynamic, causing stress waves to generate and propagate. Utilizing the signal responses from the plasma-treated PVDF nanofiber sensors, their piezoelectric coefficients were calculated.

**Figure 3:**
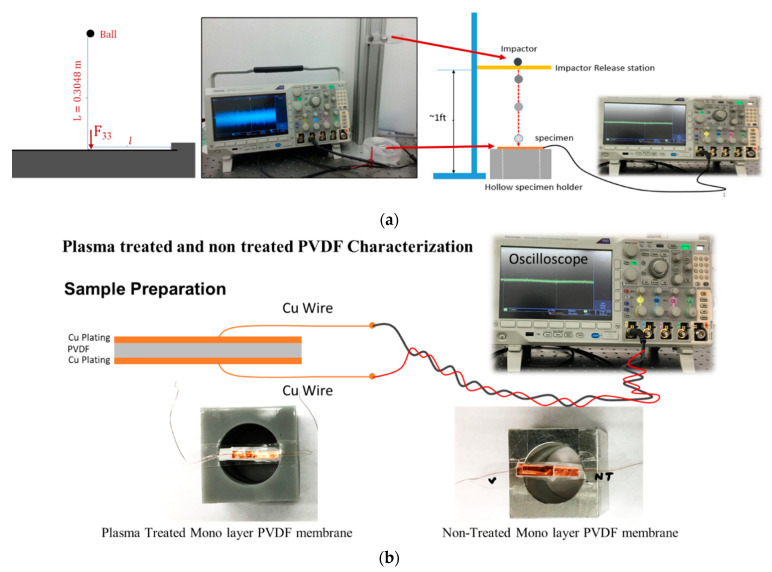
Experimental setup for evaluation of the fabricated sensors. (**a**) Schematic of the impact test and specimen with oscilloscope connection setup (**b**) Schematic of the prepared sample for the test, oscilloscope connection, and images of two-type prepared samples (plasma treated vs base PVDF).

In the experimental setup, two metallic impactor balls (½” in diameter and ¾” in diameter) with different mass weights were placed one foot above the fabricated sensors on an impactor release station. The sensors were placed on a 3D-printed PLA base with a circular hole to facilitate bending. Both side of the sensors were considered hinged. Different piezoelectric responses of treated and untreated fabricated sensors were collected. The effect of drop weight impact on the output voltage was also studied. The electrical response of the fabricated sensors was measured using an oscilloscope connected with the two electrodes placed on top and at the base of the PVDF films (Figure 3).

## 3. Results and Discussion

### 3.1. Differential Scanning Calorimetry (DSC)

The degree of crystallinity and melting points of the PVDF nanofiber membranes before and after plasma treatment were investigated using differential scanning calorimetry (DSC). The temperature range was from 0 to 350 °C with a heating rate of 3 °C min^−1^. The degree of crystallinity was estimated from the ratio of the heat of fusion of the samples to that of 100% crystalline PVDF polymer. The heat of fusion value (H_fo_) for 100% crystalline PVDF was calculated as 105 J g^−1^ [9].

**Figure 4:**
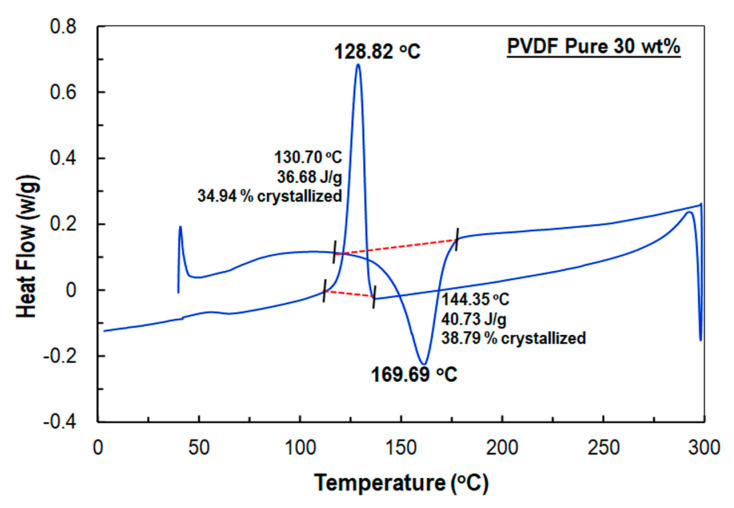
DSC thermograms of the baseline PVDF sample.

**Figure 5:**
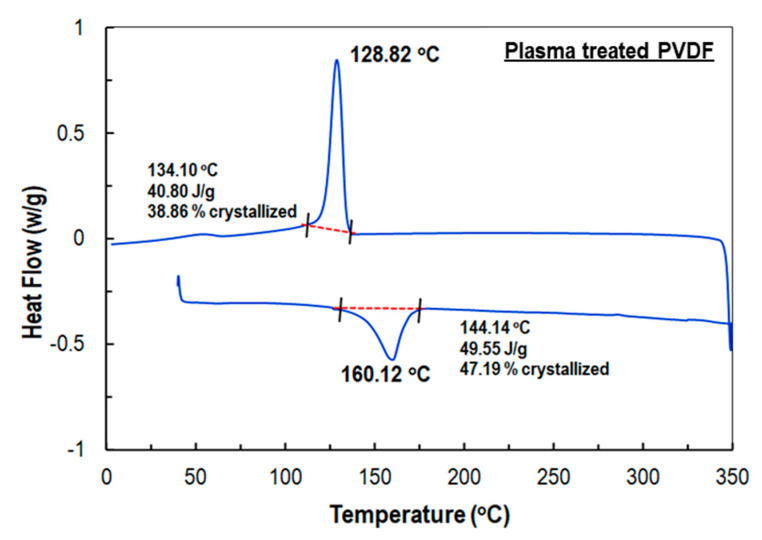
DSC thermogram of a plasma treated PVDF sample.

The melting temperature and the melting enthalpy of the PVDF nanofiber membranes with and without plasma treatment were measured as well (Table 1). As can be observed from the DSC thermograms, the melting temperature (T_m_) of standard PVDF fiber (Figure 4) (169.69 °C) was higher than the plasma-treated PVDF fibers (Figure 5) (160.12 °C). In addition, it can be seen from Figure 5 that the melting enthalpy of PVDF fibers after plasma treatment (ΔH_m_ 49.55 J g^−1^) increased compared to the untreated samples (40.73 J g^−1^). This effect was substantially more pronounced in terms of crystallinity, where the crystallinity of treated PVDF nanofiber membrane (47.19%) was significantly higher than that of the untreated one (39.79%). The results indicated that the “sub-normal” plasma could induce sufficient and noticeable change in the crystalline structure of PVDF fibers.

### 3.2. Fourier-Transform Infrared Spectroscopy (FTIR)

**Figure 6:**
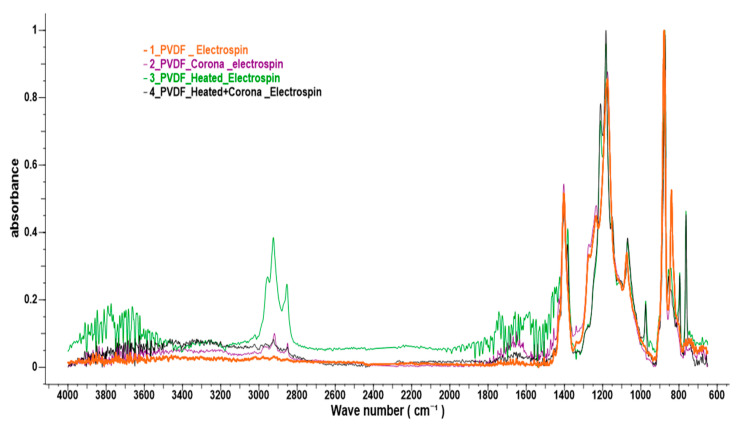
Comparison of FTIR spectrum of a pristine PVDF, Heat treated PVDF, and Plasma treated PVDF nanofiber samples.

To distinguish between the different crystalline phases, the functional groups and to evaluate the β fraction in each PVDF electrospun nanofiber membranes, FTIR spectrometry was employed. Figure 6 shows the FTIR spectra of (a) pure PVDF (b) plasma-treated (c) heated, and (d) both plasma- and heat-treated specimens.

A summary of the characteristic’s bands of the FTIR spectra are tabulated in Table 2 and Table 3. Figure 6 shows the FTIR absorbance spectra for PVDF electrospun fibers. The spectra show peaks at 726, 1339, 1383 cm^−1^ (C−H rocking), 1456, 1584, 1652 cm^−1^ (C−H bend alkanes), 736, 854 cm^−1^ (CF_2_ bonding), 1150, 1175, 1212, 1234 cm^−1^ (C−H wag (−CH_2_−X) alkyl halides), 2849, 2923 cm ^−1^ (C−H stretching), and peaks at 763, 795, 975 cm ^−1^ corresponding to α crystalline phase, and peaks at 840 and 1280 cm^−1^, which are typical vibration characteristics of the β crystalline phase.

As seen in Figure 6, a minor shift in the wavenumbers was detected for the electrospun PVDF nanofiber membrane when treated with plasma. The plasma-treated specimen showed major peaks at 1652 and 2923 cm^−1^. The spectral peaks at 1652 and 2923 cm^−1^ correlated with C–H bend alkanes stretching and C–H antisymmetric stretching vibrations, respectively. A significant increase of the intensity of the peaks and area under the curve at 1652 and 2923 cm^−1^ were detected when the specimens were heat-treated. Plasma-treated or heat-treated specimen also shows absorption peaks above 3000 cm^−1^, an indication of unsaturation: alkynyl C–H stretch. When the heated fiber was treated with plasma, no shift in wave number was observed, but the area under the curve increased, signifying the effect of plasma poling. The vibrational peaks appeared at 759–669 cm^−1^ for the plasma- and heat-treated PVDF samples, which could be attributed to C–H bending. For a heat-treated specimen, the areas under the curve at 840 and 1272 cm^−1^ (signifying β phase structure) were decreased from 53 to 37 and 30 to 29, respectively, whereas, due to the application of plasma, the areas under the curve at 840 and 1272 cm^−1^ increased from 37 to 60 and from 29 to 34, respectively. The results suggest that a transient high electric field, together with charged species interaction in plasma, can lead to an increasing ordering of the β phase compared to conventional heat-treatments.

### 3.3. Impact Test

**Figure 7:**
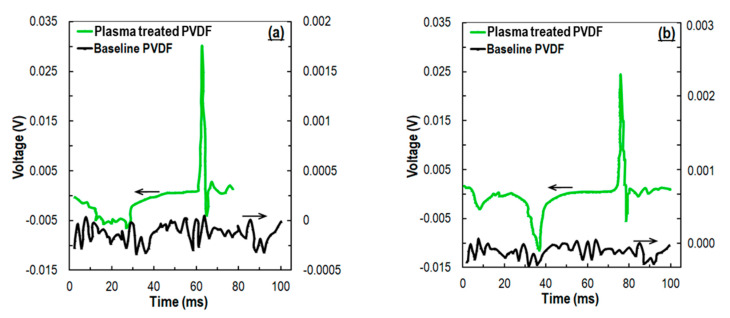
Average of 10 experiments with (**a**) big impactor ¾” (0.28 N) (**b**) small impactor ½” (0.01 N).

**Figure 8:**
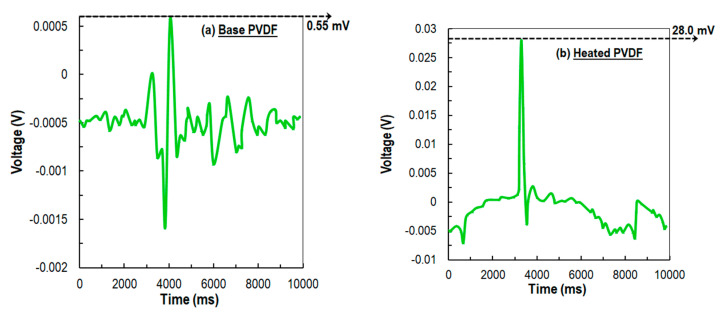

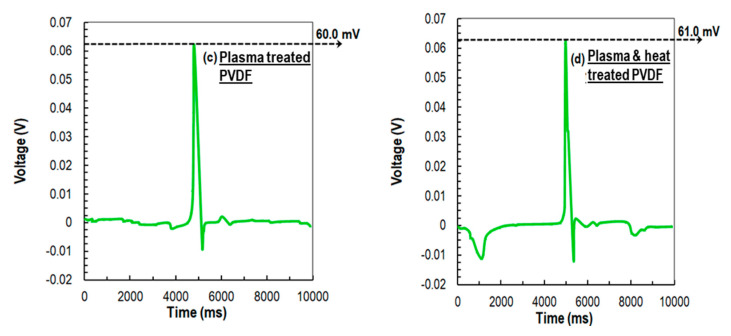
The average voltage gain characteristics from 20 experiments. (**a**) Base PVDF (**b**) heat treated PVDF (**c**) plasma treated PVDF (**d**) heated and plasma treated PVDF.

The range of output voltages may vary with the number of tests and the nature of the sensor specimens. Hence, the experiments performed herein were repeated 10 (Figure 7) and 20 (Figure 8) times, respectively, to assure repeatability of the results. It is evident from Figure 7 that the peak voltage output from the plasma-treated PVDF sample had an almost 3 times higher response than the baseline samples, i.e., ~30 and ~10 mV, respectively. The effect of the impact force on the sensor electrical response was imminent from the effective difference of output voltage when compared to the plasma-treated versus baseline PVDF sensors. In Figure 7a,b, we show the electrical responses obtained from the impacts of 0.28 and 0.01 N force. The larger impactor consistently resulted in a higher discharge voltage (~30 mV) compared to the smaller impactor (~24 mV). The voltage response further confirmed significant enhancement of the electroactive β phase content by the plasma treatment even when no other post-treatments were performed.

In the next stage of evaluation of the voltage outputs, the impact tests were repeated twenty times on each of the four different specimens as defined earlier. Surprisingly, the average voltage output from the sensors were higher when higher number of experiments (20) were conducted compared to a smaller number of drop experiments (10) reported in Figure 8. The average peak output voltage from baseline PVDF, heat-treated PVDF, plasma-treated PVDF, and plasma- and heat-treated PVDF samples were 0.55, 28, 60, and 61 mV, respectively. Finally, both the ten (Figure 7) and twenty (Figure 8) tests were found to be consistent and demonstrate a good correlation between the output voltage (although not shown). With all the observed trends, it can be concluded that the plasma-treated specimens were piezoelectrically superior to all other categories of specimens including heat-treated ones.

### 3.4. Sensor Evaluation

**Figure 9:**
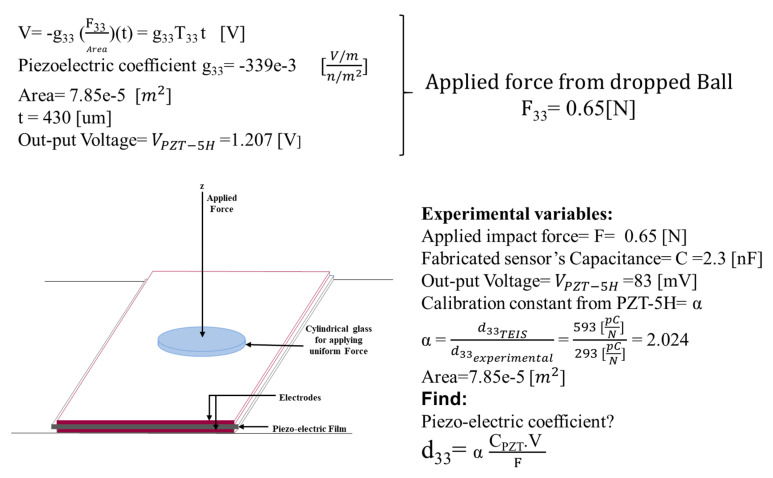
Voltage output, applied force, calibration constant and piezoelectric coefficient (d_33_) evaluation.

**Figure 10:**
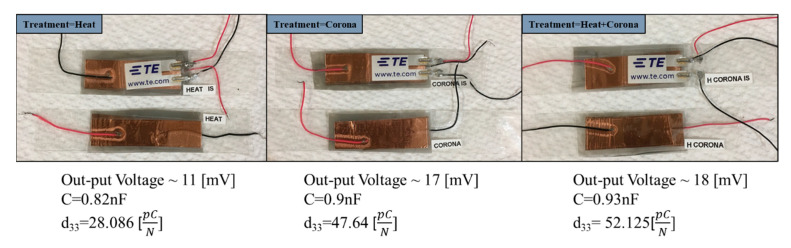
Piezoelectric Coefficient (d_33_) of three different samples.

In this section, the sensors equipped with pressure sensors were evaluated. For fabrication of each sensor, as before, a rectangular piece of electrospun PVDF film was used as an active layer that was sandwiched between two copper layers. In the next step, to have an identical experimenting procedure for all the sensors, a cylindrical glass with an area of 7.85 × 10^−5^ m^2^ was used for applying uniform force on the surface of the sensors. Using the available data sheet of the pressure sensors, the applied force was measured as 0.65 N. Considering the applied force on each of the fabricated sensors, the d_33_ piezoelectric coefficient of all the samples were measured. The process for calculating the d_33_ coefficients was demonstrated schematically in Figure 9. Figure 10 shows the results that were obtained from the heat-treated, plasma-treated, and plasma-heat-treated specimens. The d_33_ values for heat-treated, plasma-treated, and plasma-heat-treated specimens were ~28.09, ~47.64 (a ~69% increase), and ~52.12 pC/N (a ~85% increase), respectively. The capacitance was measured to be 0.82, 0.9, and 0.93 nF, respectively, for the (ii) heat-treated PVDF (iii) plasma-treated PVDF, and (iv) and plasma- and heat-treated PVDF.

## 4. Summary and Conclusions

In the present study, PVDF nanofiber membranes were fabricated by an electrospinning process and treated with a non-equilibrium plasma. The process was repeated with simultaneous plasma- and heat-treatment processes. Afterwards, sensors were fabricated to evaluate the piezoelectric response of the plasma-treated PVDF fiber. DSC test results showed that pure PVDF nanofiber was 38.79% crystalline and the melting endotherms were 40.73 J g^−1^, whereas the plasma-treated PVDF nanofiber membrane was 47.19% crystalline and the melting endotherms were 49.55 J g^−1^. The DSC results also showed a reduction of melting point from 169.69 to 169.12 °C for the plasma-treated PVDF nanofibers.

To distinguish the crystalline phase and functional groups for (a) pure, (b) plasma-treated, (c) heat-treated, and (d) plasma with heat treatment, the electrospun PVDF fiber was analyzed by FTIR spectra. For electrospun pure PVDF fiber, few peaks were found. In addition, application of plasma without any heat treatment results in a minor shift in major wave number and new peaks at 1652 and 2923 cm^−1^. After the heat treatment, the wave number shifted and new peaks were found at 1652, 2923, and 3600 cm^−1^. In addition, when the heated fibers were treated with plasma, no shift in wave number was found but the area under the curve changed, which signifies some effect of plasma poling of the fiber. By heating the fiber, the area under the curve of the β phase for wave number 840 and 1272 cm^−1^ decreased from 53 to 21 and 33 to 23, respectively. Due to the plasma treatment, the area increased from 21 to 25 for the peak 840 cm^−1^ and the area was decreased from 23 to 20 for the peak 1272 cm^−1^.

The voltage test was also conducted to corroborate the justification of the new peaks at several wave number and increment of area under the curve due to plasma treatment. The voltage response was increased to 61 mV for plasma-treated nanofiber, which was 0.55 mV for a baseline PVDF nanofiber sensor. Results from fabricated sensors showed that the piezoelectric coefficient increases to 48 pc/N (from 28 pc/N) for a plasma-treated sensor and increases to 52 pc/N (from 28 pc/N) for a plasma and heat-treated sample. The results from the DSC and FTIR testing and the improvement of the piezoelectric feature of fabricated sensors suggest that nonequilibrium plasma-treated PVDF sensors can significantly improve the output signal and sensor performances.

## Figures and Tables

**Table 1:** Comparison of crystallization point, enthalpy, and crystallization percentage of a pristine and plasma treated PVDF fibers.

Type	Crystallization Point (°C)	Enthalpy (J g^−1^)	Percent Crystallization (%)
Pure PVDF fiber	161.69	40.73	38.79
Plasma treated PVDF fiber	160.12	49.55	47.19

**Table 2:** FTIR wave number and comparison of different PVDF fiber for α and β Phase.

Type of Treatment	Changes	Phase	Effect Compare to Pure PVDF Electro-Spun Fiber
Plasma treated sample	Wave number	For β phase: 840, 1272 cm^−1^	No shift of wave number due to application of plasma on PVDF sample.
	Intensity		No change of intensity due to application of plasma on PVDF sample.
	Wave number	For α phase: 763, 795, 975 cm^−1^	No shift of wave number due to application of plasma on PVDF sample.
	Intensity		Little decrease of intensity due to application of plasma on PVDF sample.
Heat treated PVDF sample	Wave number	For β phase: 840, 1272 cm^−1^	Positive shift of wave number due to application of heat on PVDF sample.
	Intensity		Intensity decreased due to application of heat on PVDF sample.
	Wave number	For α phase: 763, 796, 975 cm^−1^	Very slight Positive shift of wave number due to application of heat on PVDF sample.
	Intensity		Intensity increased significantly due to application of heat on PVDF sample.
Both heated and plasma treated sample	Wave number	For β phase: 840, 1272 cm^−1^	No shift of wave number after the application of plasma on heated PVDF sample.
	Intensity		Intensity decreased after application of heat on PVDF sample and intensity increased further by applying plasma.
	Wave number	For α phase: 763, 796, 975 cm^−1^	Positive shift of wave number due to application of plasma for heated PVDF sample.
	Intensity		Intensity increased significantly due to application of heat on PVDF sample then it decreased little bit after application of plasma.

**Table 3:** FTIR wave number of an electro spun PVDF fiber.

Bonding			Wavenumber (cm^−1^)				Intensity			
			PVDF	Plasma PVDF	Heated PVDF	Heated + Plasma PVDF	PVDF	Plasma PVDF	Heated PVDF	Heated + Plasma PVDF
C–H rock	725–720						7			
C–X alkyl halides	850–550		748			744	8			10
		Alpha		762	764	763		11	43	44
		Alpha			796	796			24	29
C–F_2_ alkyl halides–anti symmetric stretching motion	840	Beta	839	839	840	845	58	54	37	60
C–X alkyl halides	850–550				854	854			26	29
			877	878	873	873	100	100	92	96
		Alpha				975				23
			1073	1073	1070	1070	45	36	44	42
C–H Wag (–CH_2_X) Alkyl Halides	1300–1150				1150	1151			50	50
			1173	1174	1182	1183	85	95	100	100
					1212	1211			82	81
			1234	1233			45	49		
CF–CH–CF skeletal bending motion	1274–1280	Beta	1272	1271	1275	1274	30	29	23	34
C–H rock alkanes	1370–1350			1339				16		
					1383	1383			47	45
			1404	1404	1403	1402	52	58	54	54
			1430	1430	1426	1425	15	27	28	27
C–H bend alkanes	1470–1450		1456	1456			5	56		
						1584				12
				1652	1659	1659		21	14	9
					1742				12	
C–H Stretching	3000–2850		2849	2850	2853	2853	18	10	14	15
			2923	2919	2924	2924	22	13	23	12
				2966	2955	2956		17	12	13

## Data Availability

All data generated or analyzed during this study are included in this submitted article. Data/Experimental results are presented in the manuscript and if appropriate, datasets/experimental results used and/or analyzed during the current study are available from the corresponding author on reasonable request.
